# The influence of corruption on environmental sustainability in the developing economies of Southern Africa

**DOI:** 10.1016/j.heliyon.2020.e04387

**Published:** 2020-07-07

**Authors:** Fortune Ganda

**Affiliations:** Walter Sisulu University, Faculty of Management Sciences, Department of Accounting, Butterworth Campus, Private Bag X3182, Butterworth, 4980, South Africa

**Keywords:** Corruption index, Corruption ranking, Environmental sustainability, Income, Ease of doing business, Education status, Business, Economics, Education, Environmental analysis, Environmental assessment, Environmental economics, Environmental management, Environmental pollution, Environmental science

## Abstract

This paper analyses the impact of corruption on environmental sustainability in all 16 countries in the Southern region of Africa from 2010-2017. The paper uses two proxies of corruption: the Corruption Index and Corruption Ranking. Using two econometric methods, namely, the Dumitrescu and Hurlin (2012) Granger causality test and the Generalised Method of Moments (GMM) techniques this study found largely congruent results on both causation and relationships, respectively. Firstly, the two indicators of corruption harmoniously show that corruption Granger causes the existing state of environmental sustainability in Southern African economies, and vice-versa. Moreover, in the short-run corruption was also found to worsen environmental sustainability for both regression models deployed using the two corruption indicators. In the long-term, the two measures of corruption conflicted with their findings. In this regard, though the relationship is contradicting in the long-run the corruption negative (becoming bad) effect of corruption ranking surpasses the corruption positive (becoming clean) effect of corruption index by nearly three times. This show how detrimental corruptible actions are to the natural environment. Overall, this paper consent to global reports explaining how Southern African environments are gradually deteriorating by putting corruption as one central practice causing extensive damage.

## 1 Introduction

Corruption is globally perceived as actions in which persons entrusted with power abuse it for personal benefit. Thus, Transparency International (2020) highlights that it can be categorized as grand, petty as well as political depending on the measure of money which have been lost along with the sector at hand. Machel and Coombes (2011) express that in a survey conducted in six countries from Southern Africa (Zimbabwe, South Africa, Malawi, Democratic Republic of Congo (DRC) and Mozambique) 62% of the respondents confirmed that corruption is getting worse and 56% of these respondents adds that they had to pay a bribe when they come in contact with government service agencies. Hence, Gavin (2010) adds that Southern Africa's government leading political parties are contorted in tangles as they effortlessly try to keep their privileged offices in various sectors of the economy while also seek to address corruption which inevitably has lessened their integrity along with popularity. In that case, Choruma (2018) also contributes that despite all the Southern African states being signatories of African Union (AU) Convention on Preventing and Combating Corruption (AUCPCC) of 2013 and the Southern Africa Development Community (SADC) Protocol against Corruption (SADCPC) of 2001 this challenge has increased to appalling levels and evolved to put large threat to stability, sustainable development along with socio-economic change in the region. In this vein, natural environmental issues also need immediate attention (Ganda, 2018, 2019).

In this regard, there is greater empirical literature that has established the reciprocal influence of corruption and the natural environment (Akhbari and Nejati, 2019; Sinha et al., 2019; Wang et al., 2020; Candau and Dienesch, 2017). As such, evidence supports that corruption is one of the determining variables which impacts the procedure of natural environment preservation and lessening this cause has much influence on the rate of green economic development along with sustainability. It is plain that despite countries in Southern Africa having growth prospects, these economies have not been able to maximize their fullest their policies and practices owing to corruption problems (Gavin, 2010; Choruma, 2018). Thus, when corruption affects the sectors (social, economic and political) of the country environmental sustainability initiatives will be inadequate in improving the state of the natural environment. In this vein, improving environmental sustainability has evolved into a prime focus of most empirical studies as some schools of thought argue that worsening of the natural environment is a result of inadequate resources-physical, intellectual, human and financial due to unnecessary wastage, ineffective use and poor allocation (Biswas et al., 2012; Sahli and Rejeb, 2015). Moreover, corruption has harmful impacts on environmental sustainability through minimizing the stringency of green strategies and policies as well as access to public commodities (goods and services).

The motivation behind this study is that the effect of corruption on the natural environment has led to increasing problems in the context of promoting sustainable development. The OCED (1997) express that corruption has demonstrated high impacts in less industrialised countries than high industrialised nations even though it is a common issue in both economies. World Bank (2010) also hints that African nations are predominantly constituted by weak institutional systems along with poor governance structures. In greater detail, the World Bank (2010:3) spotlights that "Corruption is embedded in the political economy of Africa. A number of studies describe the interaction between various forms of corruption and how it is intrinsically linked to the way power is exercised. In particular, when a social unit is highly diverse ethnically—as is the case in many post-independence African countries—there is likely to be suspicion and division among members, making the process of agreeing to rules for governance extremely difficult.” In this vein, the high prevalence of corruption in Africa coincides with weak performance in environmental sustainability platforms.

Thus when corruption becomes rampant in government and its agency structures and systems the natural environment is predisposed to continue being damaged owing to among others weak legislation and heightened complexity of the natural environmental matters. Thus there is no doubt that corruption is capable of disrupting government commitment, control and accountability for the natural environment (Lehman and Morton, 2017). In this vein, literature demonstrates that corruption decreases stringency of environmental regulations (in case of bias in the adoption, implementation and application stages), is the major motivator in misuse of land, ecological resources and encourages deforestation and desertification (Nyberg and Wright, 2013; Sahli and Rejeb, 2015). For instance, when a particular country experience increased levels of corruption some stakeholders can survive the impact of environmental regulations since they offer bribes to government environmental agencies. Moreover, it also makes it difficult for small and medium companies to get access to relevant environmental services and diverse green investment prospects (Lehman and Morton, 2017; World Bank, 2010). In that case, many challenges about resource depletion and natural environmental stress are born out of weak institutions and corruption fuels such situations thereby creating more problems to ecological systems and the dependent communities (Chang and Hao, 2017). The environmental costs of corruption are so hard to quantify primarily since corruption itself (owing to its characteristics and attributes) is often difficult to measure. Regrettably, for most growing economies that have large reserves of natural resources corruption is widely known as the major cause of natural environmental destruction (World Bank, 2010).

The goals of this research are hence to prove an in-depth and extensive investigation of the causation and linkages involving corruption and environmental sustainability in all the developing economies found in the Southern region of Africa. In so doing, this paper will find along with quantify these examinations by using two distinct proxies of corruption, namely the corruption index as well as the corruption ranking. It is widely agreed that corruption in many instances link to particular cases and hence difficult to quantitatively estimate its extent in any societal system. However, the form of proxies of corruption deployed by this article are globally known, computed and used by global bodies such as Transparency International to look at the status of corruption. Moreover, other aims of this article is to; offer a detailed theoretical perspective of how corruption influence environmental sustainability, and give a synopsis of the study results and then elaborate on the implications such findings has for environmental policy of the studied countries in efforts towards improving environmental sustainability.

The contributions of this research are manifold. First, there are limited studies with evidence that have evaluated the effects of corruption on environmental sustainability in developing countries of Africa yet these economies are known as the most corrupt states in global rankings and statuses. Secondly, to the best of my knowledge, this paper is the first one to employ two different proxies of corruption to get their effects on environmental sustainability. In doing so, the paper will examine the congruence and contradictory nature of the findings to effectively acquire an accurate and strong view of the linkages, and causation. Third, despite this interest about corruption, it is still not clear if it causes and/or relates to environmental sustainability and vice-versa since empirical studies still generate mixed findings. As such, this paper deploys two measures of corruption to the same group of countries to find out the scenarios. Fourth, this article is unique as it uses two advanced econometric procedures to first understand causation and secondly comprehend associations involving corruption and environmental sustainability. To show causation this paper adopts the Dumitrescu and Hurlin (2012) Granger causality tests which is one of the most recent techniques. Thus, this approach is suitable for heterogeneous panelized data frameworks which consider individual unit fixed effects. The paper will employ this method based on a bootstrap procedure since that process solves cross-sectional dependence challenges. To set-up relationships this article employs a two-step GMM approach and generally, the GMM technique considers country-specific effect along with simultaneity biases. In the two-step procedure, GMM acquires parameter estimates subject to the initial weight matrix, generates a new weight matrix dependent on those results and then re-calculate the parameters built on that weight matrix. The benefits of the two-step technique is that the number of equations, as well as parameters in the non-linear GMM step, do not increase with the number of perfectly estimated regressor factors hence it is very effective in improving consistency, efficiency and the power of related tests than one-step GMM and the difference GMM. The two-step GMM outputs are based on both short-run and long-run contexts.

This research is organised as follows. Section 2 provides a literature review of the linkages involving corruption and environmental sustainability. Section 3 describes the study method and data of this paper. Section 4 discloses and analyze the findings. Section 5 discusses the implications of the study. Section 6 concludes.

## 2 Literature review

A plethora of literature on corruption and environmental sustainability has received greater attention in recent years in different global contexts. Table 1 below presents some of the recent empirical literature on this subject.

**Table 1:** Showing empirical studies and their results.

Author(s)	Country(s)	Period	Variables	Methodology	Result (s)
Akhbari and Nejati (2019)	61 Countries	2003–2016	Carbon Emissions (CO_2_); Gross Domestic Product (GDP); Primary Energy Consumption (E); Trade (T); Corruption Index (CORI); Human Development Index (HDI) and Urban Population Growth (UPG)	Panel threshold model.	In developing economies corruption increases emissions while in developed countries corruption no longer influences carbon emission levels.
Wang et al. (2018)	Brazil, Russia, India, China and South Africa (BRICS)	1996–2015	CO_2_; GDP; T; CORI; Population Growth (PG); Urbanisation (U).	Partial Least Square Regression Model.	The moderating role that corruption play is critical on the association involving GDP and CO_2;_ U and CO_2_; plus T and CO_2_. Corruption management lessens emissions.
Zhang, Jin, Chevalli, and Shen (2016)	19 Asia-Pacific Economic Cooperation (APEC) economies	1992–2012	CO_2_; GDP; T; CORI; Population (P); Urban Population (UP); PG; Inflation Rate (I); Democratic Accountability (DA); E.	Panel quantile regression Approach	The negative impact of corruption in APEC lower emission countries was noticeable but that influence was not significant in higher emission economies. Inverted U-shaped Environmental Kuznets Curve (EKC) between corruption and CO_2_ is valid. Corruption possess both a negative direct impact on CO_2_ and positive indirect impact by its impact on GDP.
Sinha, Gupta, Shahba, and Sengupta (2019)	BRICS; and the Next 11 countries.	1990–2017	CO_2_; GDP; E; T; U: CORI and P.	Generalized Method of Moments (GMM)	Corruption promotes environmental damage through lessening the positive effect of green energy use on environmental quality along with heightening the negative influence of non-renewables deployment.
Arminen and Menegaki (2019)	67 high-income and upper-middle-income countries.	1985–2011	CO_2_; GDP; E; T; Industrialisation (IDN: CORI, Physical Capital Stock (PCS); Human Capital (HC) and Temperature (TC).	Simultaneous equations framework	Climate and weather variables are more critical factors influencing E and CO_2_ when compared to corruption. Moreover, transformations in institutional quality (proxy is corruption) generate less effect on energy and environmental policy.
Wang et al. (2020)	China	2006–2015	Ecological efficiency level (EFL); Resource misallocation (RM); Corruption cases (CC); Government regulation (GR); Logistics level (LL) and Industrial structure (IS).	GMM	Corruption, as well as misallocation of resources, possess detrimental effects on ecological efficiency. Corruption also intensifies resource misallocation thereby further lessening ecological efficiency.
Dincer and Fredriksson (2018)	48 United States of America (USA) states	1977–1994	Stringency of Law, Corruption Index; Trust; Income; Energy Prices; Land prices; Percentage of legal services and Education	GMM	Increased corruption minimizes the strictness of natural environmental policies in cases of low trust degree but that impact reduces and also develop to be positive in high levels of trust.
DiRienzo and Das (2019)	180 countries	2018	Country Environmental Quality & Performance; Corruption; Women in vital political positions; Economic Development; Education; Income Inequality; Rule of Law; Democracy; Economic Freedom	Multi-step Regression Frameworks	Women in influential political positions have a positive influence on environmental results although this impact is embedded by their effect on lessening corruption.
Chen et al. (2018)	30 Chinese provinces	1998–2012	Environmental pollution; Environmental regulation; Shadow Economy; Government corruption; GDP; T; Education level; Value added by Industry (VI); Population density; Research and Development (R&D) strength.	GMM	Increases in the number of corrupt officials results in a less effective and/or weakened environmental legislation that eventually generates high illegal production along with total pollutant emissions.
Cole (2007)	94 countries	1987–2000	Sulphur Dioxide; Carbon emissions; GDP(Income); Pollution; Corruption	Regression analysis involving the use of instrumental variables and sensitivity analysis.	Corruption has a direct positive impact on both Sulphur Dioxide and Carbon emissions although indirect impacts were determined to be negative and large. As such, the aggregate impact of corruption on environmental quality is negative for the majority of countries except the high-income countries where it tends to be positive.
Meehan and Tacconi (2017)	Indonesia	January 2011–November 2011	Land-use planning; Awarding concession and permits to utilize forests; Monitoring and enforcement of regulations	Field Research	The effects of a diverse range of corruption on forest management can be direct, indirect, complicated and also negligible. Hence, anti-corruption initiatives should focus more on particular forms of corruption which are possibly adding to deforestation along with forest degradation.
Sahli and Rejeb (2015)	21 MENA countries	1996–2013	GDP; Exports(X); Imports(M); T; VI; and population density; Carbon emissions; Corruption level.	Panel Dynamic Regression models	A direct positive effect of corruption on both emissions and GDP is present. The Environmental Kuznets Curve (EKC) is also a valid present.
Wu et al. (2017)	China	2007–2014	Total factor productivity (TFP); Government Expenditures; Corruption level; Industrial structure; Foreign Direct Investment; Financial Development	Dynamic spatial autoregressive model and the Panel threshold model	Heightened corruption incidences have a direct reducing impact on regional total factor productivity. The influence of government expenditures (administrative service, investment development and safeguard governance) on total factor productivity possess only one corruption threshold.
Candau and Dienesch (2017)	International European-controlled enterprises	2007–2010	Bilateral trade; Corruption Index; Inflation; Environmental Regulation; GDP;	Fixed Effects Regressions	Corruption reduces environmental standards
Biswas et al. (2012)	100 countries	1999–2005	Sulfur Emissions; GDP; Corruption index; T; Energy Efficiency; U; P; Shadow Economy	Fixed Effects Regressions	A shadow economy and level of emissions are largely influenced by the level of corruption.
Krishnan et al. (2013)	105 countries	2004–2008	E-government maturity; Corruption; GDP; Carbon emissions; UP; Exports; Political Stability; Regional Difference	Structural equation modelling (SEM) analysis	E-government maturity does not add to GDP and environmental damage although its significance is visible indirectly through its effects on corruption.
Hao et al. (2020)	China 30 provinces	2005–2016	Green total factor energy efficiency; Resource Misallocation; Corruption	Spatial econometric methods, Panel threshold model.	Capital misallocation in local community (though not significant) and neighbouring area (significant) develop negative links with Green total factor energy efficiency. Corruption does not influence green total factor energy efficiency, but escalates inhibiting impacts both labour and capital resource misallocation.
Wawrzyniak and Doryń (2020)	93 countries	1995–2014	Emissions; Corruption; Government effectiveness; Economic growth.	GMM	The moderating role as regards to control of corruption on emissions and economic growth relationship was not found.
Zandi et al. (2019)	6 ASEAN countries	1995–2017	Corruption; Military Expenditure, Democracy; Emissions.	Fully Modified Ordinary Least Square (FMOLS) and Dynamic Ordinary Least Square (DOLS)	Corruption has a positive and significant effect on environmental quality.
Yahaya et al. (2020)	8 Sub-Saharan African countries	2000–2014	Financial Development; Corruption; Environmental degradation	FMOLS	Corruption, along with interaction with financial development has a positive and significant influence on environmental degradation

The past literature in Table 2 employed diverse measures of institutional quality to check its effect on the natural environment. In this article, I adopt two of the major proxies of corruption to assess how they simultaneously influence environmental sustainability under similar conditions (such as, similar countries, period, econometric techniques, and the same control variables). Against the background of developing countries in the Southern African region's political and economic frameworks the regions, natural environment landscape is consistently deteriorating and this paper is important to prove how corruption is impacting on environmental sustainability in this region.

**Table 2:** Showing detailed description of variables.

Variable	Definition	Unit	Source
CORI	Corruption Index	Points out of 100 yearly. Thus, 100 (very clean) to 0 (highly corrupt) (Transparency International, 2020)	Transparency International.
CORRA	Corruption Rank	Position relative to other global countries that are included in the index (Transparency International, 2020)	Transparency International.
GDP	Economic Growth	Gross Domestic Product (GDP) per capita	World Bank
EDB	Ease of Doing Business	Index. Higher ranking (low numerical estimate) show a better environment for doing business, and vice-versa (World Bank Group, 2020)	World Bank.
EDU	Adjusted savings: education expenditure. *NB. This variable is a proxy of the state of education.*	Percentage of GNI (Gross National Income)	World Bank
ENS	Adjusted net savings, excluding particulate emission damage. *NB. This variable is a proxy of environmental sustainability*	Percentage of GNI (Gross National Income)	World Bank

Note: [1] The Adjusted net savings, excluding particulate emission damage, indicates the dependent variable. The remaining variables are all explanatory variables. [2] The data was analysed in logarithm form to ensure compactness since most of the variables in this study are non-linear.

## 3 Methodology

This section presents and discusses sections on the data, panel causality tests, cross-section dependence test and the Generalised Method of Moments (GMM) approach.

### 3.1 Data

Data employed in this study is extracted from Transparency International and the World Bank over the period 2010 to 2017. The sample of countries which are the focus of the study are all the 16 Southern African countries. These are, namely, Angola, Botswana, Comoros, Democratic Republic of Congo, Eswatini, Lesotho, Madagascar, Malawi, Mauritius, Mozambique, Namibia, Seychelles, South Africa, Tanzania, Zambia and Zimbabwe. The full description of the variables utilised in this study is presented in Table 2.

### 3.2 Panel causality test

This paper makes use of the panel causality test introduced by Dumitrescu and Hurlin (2012). Panel causality tests will aid the paper to understand causality. This form of tests is an unambiguous framework of Granger (1969) non-causality test version for heterogeneous panelised data structures with fixed coefficients. Also, it considers two heterogeneity classifications (heterogeneity of the regression equation employed to investigate Granger causality along with the heterogeneity of the causality associations).

Thus, initially I take into account the following framework (Equation 1):

[1] $$y_{i,t}=\alpha_{i}+\sum_{k=1}^{K}\gamma_{i}^{\left(k\right)}y_{i,t-k}+\sum_{k=1}^{K}\beta_{i}^{\left(k\right)}y_{i,t-k}+\varepsilon_{i,t}\;i=1\text{,}2\text{,.......}N\text{: }t=1\text{,}2\text{,.......,}T$$

In this case, *x* and *y* represents two stationary variables identified for *N* individuals in *T* periods. $\beta_{i}={\left(\beta_{i}^{\left(1\right)},\ldots ..,\beta_{i}^{k}\right)}^{t}$ along with individual effects $\alpha_{i}$ are understood to be fixed in the time dimension specification. Further, the lag orders of *K* are assumed to be homogenous for the complete cross-section of the panelised data under the survey. Besides, autoregressive parameters $\gamma_{i}^{\left(k\right)}$, as well as $\beta_{i}^{\left(k\right)}$ that are the regression coefficients, are permitted to be different across groups.

This test approach puts forward that the null hypothesis is assumed to have no causality association for any units available (*x* and *y*) in the panel data. Therefore if $H_{o}$ is rejected the study will state that causality from *x* and *y* exists. It also follows that *x* and *y* can be interchanged to investigate causality in the other direction (bidirectional causality) also referred to as feedback impacts.

This assumption that is often identified as the Homogenous Non-Causality (HNC) hypothesis and is explained as below:

$$H_{o}:\beta_{i}=0,\forall i=1,\ldots \ldots .N$$

The alternative hypothesis is recognized as the Heterogeneous Non-Causality (HENC) hypothesis. Therefore, under the HENC two subcategories of cross-section units are permitted.

On one hand, there is a causality association from *x* to *y* for the initial model, although it is not sufficiently founded upon the same regression framework. On the other hand, the second subcategory highlights that there is no causality association from *x* to *y.* We are taking into account a heterogeneous panelised data framework constituting fixed coefficients (in time) as regards to this group. The alternative hypothesis is hence presented as:

$$\begin{array}{l} H_{1}:\beta_{i}=0,\;\forall i=1,\ldots \ldots .N_{1}\\ \beta_{i}\neq 0,\;\forall i=N_{1}+1,.......N \end{array}$$

It is assumed that $\beta_{i}$ can differ across groups plus there are $N_{1}$ (Nindividual procedures that have no causality from *x* to *y.* It is also that $N_{1}$ is not known but it permits the condition 0 ≤ $N_{1}$/ N < 1.

As such, the average statistic $W_{N,\;T}^{HNC}$ which is associated with the null HNC hypothesis is proposed as below:

[2] $$W_{N,T}^{HNC}=\frac{1}{N}\cdot \sum_{i=1}^{N}W_{i,T}$$

It follows that $W_{i,\;T}$ illustrates the individual Wald statistics as regards to the i^th^ cross-section unit to match the individual test hypothesis $H_{o}:\beta_{i}=0$.

Let $Z_{i}$ = [*e*:$\;Y_{i}$:$\;X_{i}$] be the (*T*, 2*K* + 1) matrix, in which *e* shows a (*T*,1) unit vector and $Y_{i}$ = [$y_{i}^{\left(1\right)}$: $y_{i}^{\left(2\right)}$:……:$\;y_{i}^{\left(K\right)}$], $X_{i}$ = [$x_{i}^{\left(1\right)}$: $x_{i}^{\left(2\right)}$:……:$\;x_{i}^{\left(K\right)}$]. $\theta_{i}$ = ($\alpha_{i}\gamma_{i}^{'}\beta_{i}^{'}$) represents vector of parameters of the framework. Moreover, I let *R* = [0:$1_{K}$] be a (*K*, 2*K* + 1) matrix.

For every *i* = 1,…..*N*, the Wald statistical estimate $W_{i,T}$ to match to the individual tests $H_{o}$: $\beta_{i}$ = 0 is elaborated as follows:

[3] $$W_{i,T}={\ddot{\text{θ}}}_{i}^{'}R^{'}{\left[{á}_{i}^{2}R{\left(Z_{i}^{'}Z_{i}\right)}^{-1}R^{'}\right]}^{-1}R{\ddot{\text{θ}}}_{i}$$

Concerning the hull hypothesis of non-causality, every Wald statistic value links up to a chi-squared distribution that has *K* degrees of freedom for *T* →∞.

$$W_{i,T}\to \chi^{2}\left(K\right),\forall i=1,\ldots \ldots .N$$

The standardized test statistic estimate $Z_{N,\;T}^{HNC}$ for *T, N* →∞ is presented as:

[4] $$Z_{N,\;T}^{HNC}=\surd \frac{N}{2K}\left(W_{N,\;T}^{HNC}-K\right)\to N\left(0,1\right)$$

As well, the standardised test estimate ${Ž}_{N}^{HNC}$ for fixed T samples is outlined as:

[5] $${Ž}_{N}^{HNC}=\surd \frac{N}{2K}\cdot x\frac{\left(T-2K-5\right)}{\left(T-K-3\right)}\cdot x\;\left[\frac{\left(T-2K-3\right)}{\left(T-2K-1\right)}\cdot W_{N,T}^{HNC}-K\right]\to N\left(0,1\right)$$

Thus, in equation (4) and equation (5), $W_{N,T}^{HNC}$ = (1/N)$\;\sum_{i=1}^{N}W_{i,T}$.

To summarise, the statistic values explained above the Granger causality process output reports the values obtained for *W*(W-bar), Z (Z-bar), and *Ž*(Z-bar tilde). Dumitrescu and Hurlin (2012) express that if *N* is large but *T* is small then *Ž* should be favoured. Therefore, in this paper, I test for Granger causality in the panel set by employing Dumitrescu and Hurlin (2012) *xtgcause* command in the Stata package using specifically the bootstrap procedure as it is also able to solve problems associated with cross-sectional dependence (Lopez and Weber, 2017). Still, on the subject of cross-sectional dependence, this paper conducts a second-generation panel unit-root test most possibly the one suggested by Pesaran (2007).

### 3.3 Cross-section dependence test

As regards to panel data analysis process, before ascertaining stationarity of the series, there is a need to test the framework to determine if cross-sectional dependence is there or not. Therefore, the hypotheses set-up to analyze cross-sectional dependence are presented as below:

$H_{o}$: Cross-sectional dependence.
$H_{1}$: No cross-sectional dependence.

If $H_{o}$ is rejected, a first-generation unit root test will be employed but if $H_{o}$ is accepted a second-generation unit root test process will be deployed. In addition, cross-sectional dependence in the framework is also supported with an understanding of *N > T* along with *T > N*. It is known that Pesaran (2004) Cross Sectionally Dependency Lagrange Multiplier (CDLM) test is normally applied in cases where *N > T* but the Breusch and Pagan (1980) CDLM 1 test as well as the Pesaran (2004) CDLM 2 test is usually employed when *T > N* condition. In this article, I test cross-sectional dependence under conditions *N > T* for the Southern African countries (*N =15*) over the period 2010–2017 (*T= 8* years).

As such, Pesaran (2004) had employed the CDLM test for studies with panels that have *N* →∞ as well as *T* →∞. Thus Pesaran test statistic is ascertained as follows:

[6] $$\text{CDLM test}=\surd \frac{1}{N\left(N-1\right)}\sum_{i=1}^{N-1}x\sum_{i=1}^{N}x\;\left(T\rho_{ij}^{2}-1\right)$$

Where $\rho_{ij}^{2}$ is the sample value of the pair-wise correlation of the residuals. As mentioned earlier Pesaran (2004) has utilized the CD test for the investigation of cross-sectional dependence when *N* is larger than *T*. This type of test rest on the aggregate value of correlation coefficients involving cross-sectional residuals. Therefore, the test statistic is prepared as follows:

[7] $$\text{CD}=\surd \frac{2T}{N\left(N-1\right)}\sum_{i=1}^{N-1}x\sum_{i=1}^{N}x\rho_{ij}$$

### 3.4 Generalised Method of Moments (GMM)

The paper will initially test the presence of the Random Effect or Fixed Effect in the framework before implementing the dynamic GMM procedure (ideal for this paper to show the direction of relationships on both short-run and long-run). In this regard, the Hausman tests will also be applied and inevitably rejects the null hypothesis which states that the Random Effect (RE) model is most suitable in favour of the alternative- Fixed Effect (FE), vice-versa. The GMM approach is an advanced econometric tool that is largely known for generating relatively efficient estimators. In this context, the rigour plus efficiency of any finite sample is scrutinized using Arellano–Bond and the Blundell–Bond GMM estimation techniques that take into account the presence of heteroskedasticity owing to the dynamic character of data involved as well as endogeneity (Arellano and Bover, 1995; Blundell and Bond, 1998). For this paper, the GMM package is utilized to manage the dynamics, heteroskedasticity, and endogeneity found in the regression frameworks. Thus the regression models of this paper are presented as follows:

[8] $$ENS_{it}=\alpha_{1}+\alpha_{2}ENS_{it-1}+\alpha_{3}CORI_{it}+\alpha_{4}GDP_{it}+\alpha_{5}EDB_{it}+\alpha_{6}EDU_{it\;\;}\;+\varepsilon_{it}$$

[9] $$ENS_{it}=\alpha_{1}+\alpha_{2}ENS_{it-1}+\alpha_{3}CORRA_{it}+\alpha_{4}GDP_{it}+\alpha_{5}EDB_{it}+\alpha_{6}EDU_{it\;\;}+\;\varepsilon_{it}$$

where, *i* represents the country (*i* = 1,…*N*) while *t* shows the time period (*t* = 1…*T*). ENS illustrates is a measure/indicator of environmental sustainability. ${\text{ENS}}_{it-1}$ is the lagged dependent factor of environmental sustainability. EDU demonstrates the proxy for the state of education level contexts. CORI represents the corruption index. GDP shows the economic growth of the country. CORRA is the corruption rank of the country. EDB is a variable which outlines the ease of doing business.

Therefore, when applying GMM; transformations in one explicatory factor influence dependent variables although it regulates over some time to that effect as it approaches its long-term equilibrium. Thus, the GMM handles the entire system of equations as regards to panel data plus extension to panel study and not just a single equation. As such, the dynamics of the data set are effectively monitored by this method through enveloping the cross-sectional variances along with including differenced lagged estimates as instruments making the estimators unwavering and steady.

This paper will make use of the system GMM estimator rather than the difference GMM (Arellano and Bover, 1995; Blundell and Bond, 1998). Arellano and Bover (1995) along with Blundell and Bond (1998) highlights that the difference GMM act mediocre and results to big sample biases in cases where explanatory factors are consistent over time and the non-appearance of information as regards to the focus factors in the level classification can lead to loss of large components of total variance in the panel data. Bond et al., (2009) confirm that the system GMM estimators are produced when the factors in their differences form are instrumented with lags that fit their levels, while factors in levels are instrumented with lags that pertains to their respective differences. About the system GMM even though the levels of the independent factors are ideally correlated with country particular fixed effect, the variances are not correlated. Moreover, time dummies can be added to manage time-specific impacts and to get rid of cross-sectional dependence in the panel data along with country or unit dummies can be employed to handle country-specific effects or unit impacts.

## 4 Results and discussion

This section presents and outlines the findings of the paper.

Table 3 illustrates the statistical attributes of the variables shown in the regression framework [Eq. 1 and/or 2]. It is clear that each variable shows diverse distribution patterns which are absolutely distinct. Hence, use of the Ordinary Least Squares (OLS) regression technique may generate biased outcomes. As such, the use of Granger Causality tests will help to show causality and the employment of the GMM approach will find the relationships of these variables.

**Table 3:** Statistical summary of variables.

Variable	Min.	Std. Dev.	Max.	Mean	Skewness	Kurtosis
ENS	-40.98137	13.53447	32.13046	1.822031	-0.2728881	2.996398
CORI	15	12.73515	65	36.34028	0.4396753	2.12016
CORRA	28	43.13015	168	97.61806	0.103078	1.680714
GDP	322.4	3633.37	14014.9	3620.018	1.032786	3.044089
EDB	17	45.38501	187	119.0694	-0.4977871	2.334354
EDU	2.75 × 10^7^	2.75 × 10^9^	2.41 × 10^10^	1.92 × 10^9^	3.541297	14.32835

It is widely accepted that there is a need to investigate the stationary level of each variable under study as they are normally viewed to be non-stationary. Out of the many panel unit root tests suggested in the available literature, this paper employed the Fisher ADF test, Harris-Tzavalis test, and the Im-Pesaran-Shin (IPS) test. These particular forms of tests have diverse roots plus they can reduce homogeneity challenges. The null-hypothesis of non-stationary is analyzed for each variable in the paper and the results are presented in Table 4 above. All variables are found to be stationary at their respective first difference level which permits the study to estimate the regression coefficients.

**Table 4:** Panel Unit test results.

Variable	At Level			At 1^st^ Difference		
	Fisher ADF statistic	Harris-Tzavalis Statistic	Im-Pesaran-Shin Statistic	Fisher ADF statistic	Harris-Tzavalis Statistic	Im-Pesaran-Shin Statistic
ENS	5.9469 (0.0000)∗∗∗	-4.1391 (0.0000)∗∗∗	-1.5573 (0.0597)∗	16.3045 (0.0000)∗∗∗	-10.3690 (0.0000)∗∗∗	-4.0283 (0.0000)∗∗∗
CORI	2.5080 (0.0061)∗∗∗	-1.7387 (0.0410)∗∗	-1.1446 (0.1262)	7.5606 (0.0000)∗∗∗	-8.4414 (0.0000)∗∗∗	-3.0383 (0.0012)∗∗∗
CORRA	-0.9693 (0.8338)	-2.1784 (0.0147)∗∗	-0.3420 (0.3662)	9.0693 (0.0000)∗∗∗	-9.8421 (0.0000)∗∗∗	-3.3103 (0.0005)∗∗∗
GDP	6.6090 (0.0000)∗∗∗	2.4188 (0.9922)	-0.2336 (0.4076)	1.9188 (0.0275)∗∗	-9.4802 (0.0000)∗∗∗	-0.3681 (0.3564)
EDB	-0.9574 (0.8308)	-0.0569 (0.4773)	-0.2481 (0.4020)	8.6532 (0.0000)∗∗∗	-9.6338 (0.0000)∗∗∗	-3.0459 (0.0012)∗∗∗
EDU	0.9148 (0.1801)	-4.3535 (0.0000)∗∗∗	-0.7449 (0.2282)	2.4899 (0.0064)∗∗∗	-3.6809 (0.0001)∗∗∗	-2.1549 (0.0156)∗∗

Notes: ∗∗∗: ∗∗ and ∗ indicate that the coefficients are significant at the 1%, 5% and 10% level of significance, respectively.

However, recent literature postulates that there is a possibility that panel data sets can be dependent on cross-sections. As such this study conducted a cross-section dependence (CD) test and the results are shown in Table 5 below. In this case, the outcomes demonstrated in Table 5 confirm that the null hypothesis ($H_{o}$: Cross-sectional dependence) is rejected and the alternative hypothesis ($H_{1}$: No cross-sectional dependence) is accepted. Hence, the rejection of the null hypothesis implies that any changes and/or a specific shock for any variable of a country that is part of the study do not produce changes in that particular variable in the remaining countries that are part of the panel data set.

**Table 5:** Showing CD tests results.

Variable	CD test	p-value
ENS	-0.35	0.032∗∗
CORI	21.79	0.000∗∗∗
CORRA	5.65	0.000∗∗∗
GDP	2.85	0.004∗∗∗
EDB	1.47	0.018∗∗
EDU	10.22	0.000∗∗∗

Notes: ∗∗∗: ∗∗ and ∗ indicate that the coefficients are significant at the 1%, 5% and 10% level of significance, respectively.

### 4.1 Findings about correlations of the variables

In this section of the paper, I discuss the correlation involving all the main variables of this study. High correlations are normally values that are significantly close to -1 and/or +1 and therefore show multi-collinearity which inevitably requires estimation of the variance inflation factor (VIF). If VIF is more than 10, high multi-collinearity is valid and may greatly affect results outputs of ordinary least square regression estimates (Hair et al., 1995; Wold et al., 1984). As illustrated in Table 6 all independent variables show a VIF less than 10 except for the dependent variables (Corruption Index (CORI) and Corruption Ranking (CORRA) since their VIF is greater than 10). It is vital to note that multicollinearity normally affects independent variables and not dependent variables (Hair et al., 1995; Wold et al., 1984) and this study is not affected by multicollinearity. Furthermore, to account for the multi-collinearity challenges this paper will also not substantiate findings generated by the OLS regression but employ the GMM approach along with the Granger causality approach.

**Table 6:** Showing correlation matrix and multicollinearity.

Correlation Matrix							Multicollinearity		
Variable	ENS	CORI	CORRA	GDP	EDB	EDU	R^2^	Tolerance	VIF
ENS	1						0.4828	0.5172	1.93
CORI	0.5653	1					0.9471	0.0529	18.89
CORRA	-0.5944	-0.9636	1				0.9419	0.0581	17.23
GDP	0.1958	0.7724	-0.7199	1			0.7097	0.2903	3.44
EDB	-0.4455	-0.8314	0.8520	-0.6917	1		0.7758	0.2242	4.46
EDU	-0.0349	0.1054	-0.1433	0.2681	0.2010	1	0.2571	0.7429	1.35

**Figure 1:**
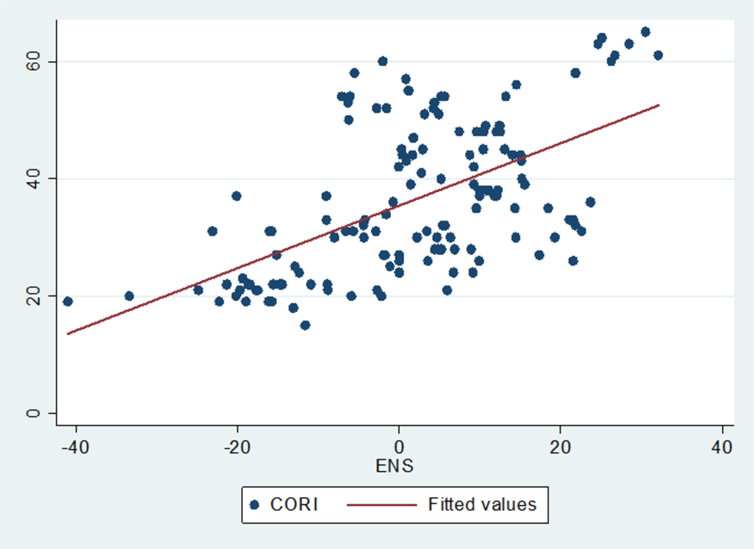
Showing the relationship between the Corruption Index (CORI) and environmental sustainability.

**Figure 2:**
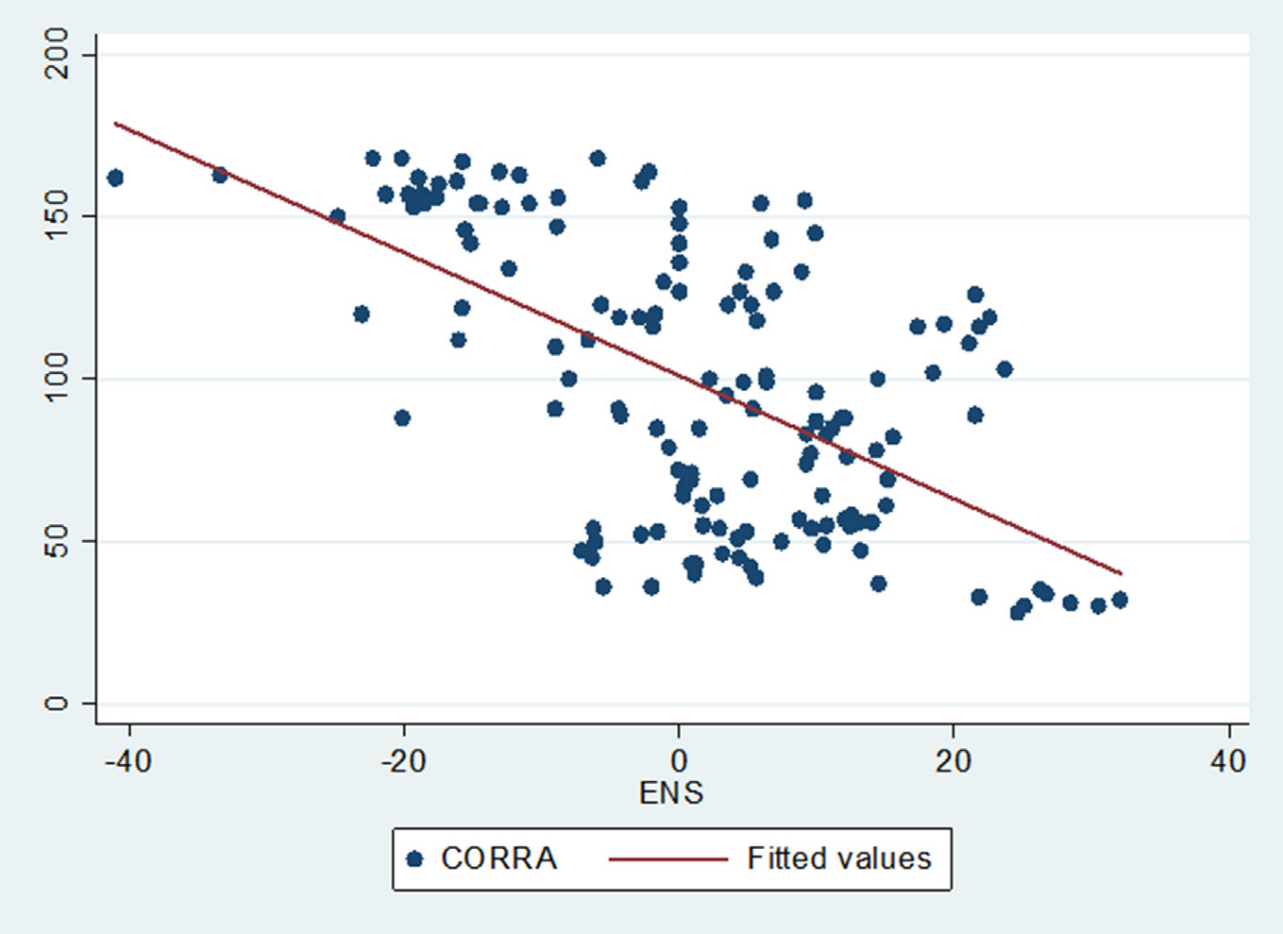
Showing the association between Corruption Ranking (CORRA) and environmental sustainability.

**Figure 3:**
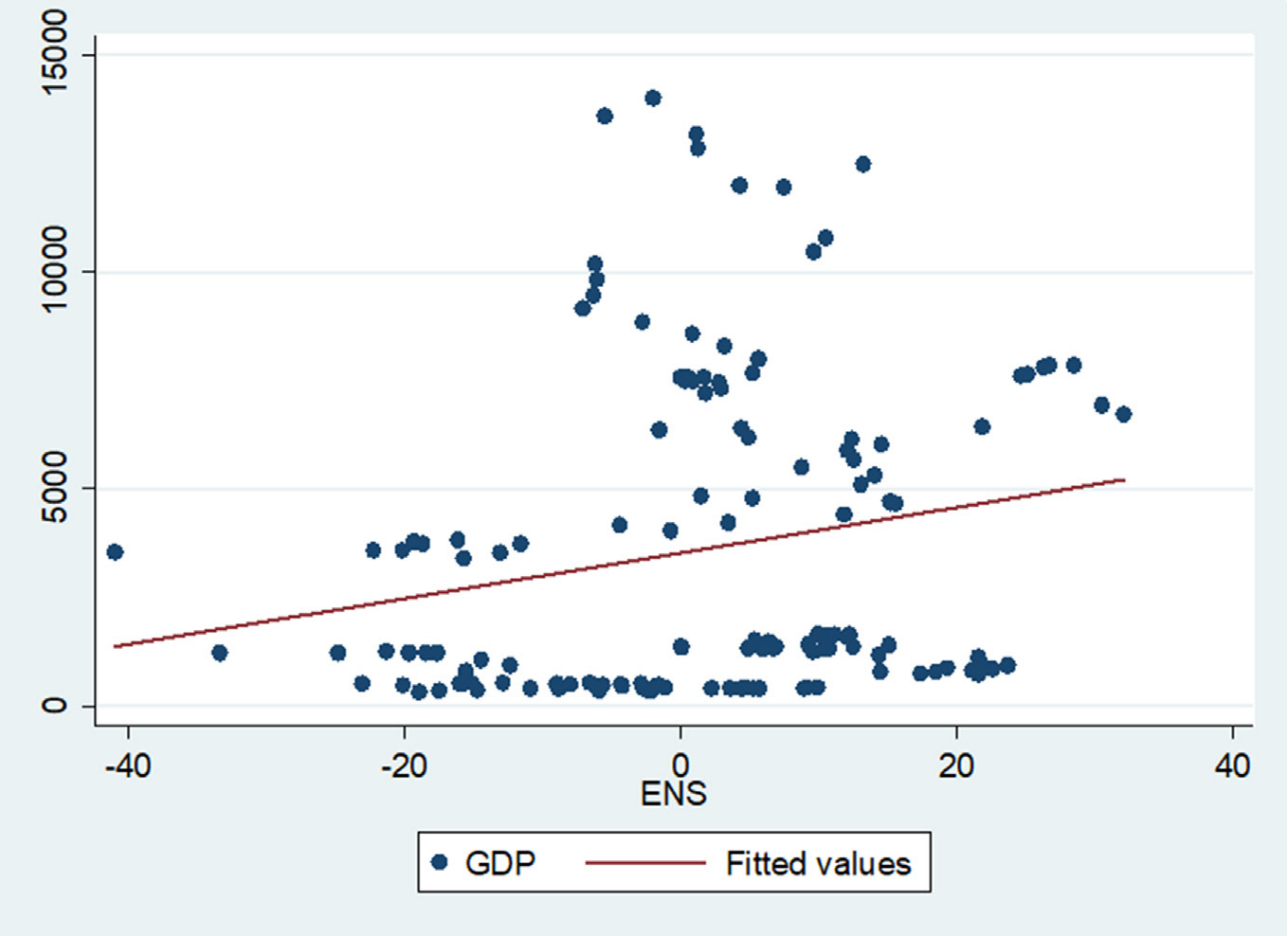
Illustrating the relationship between income (GDP) and environmental sustainability.

**Figure 4:**
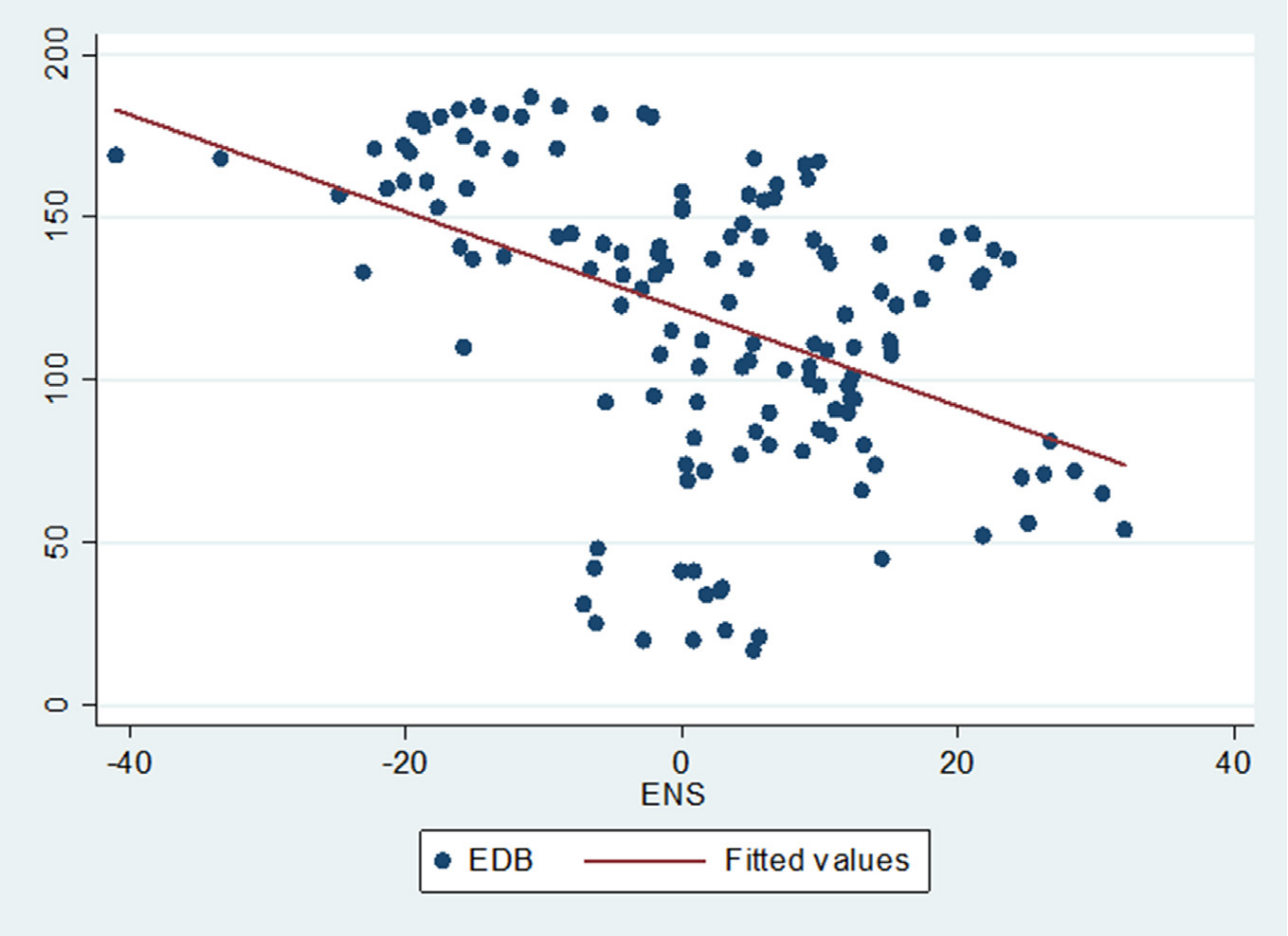
Showing the relationship between ease of doing business and environmental sustainability.

**Figure 5:**
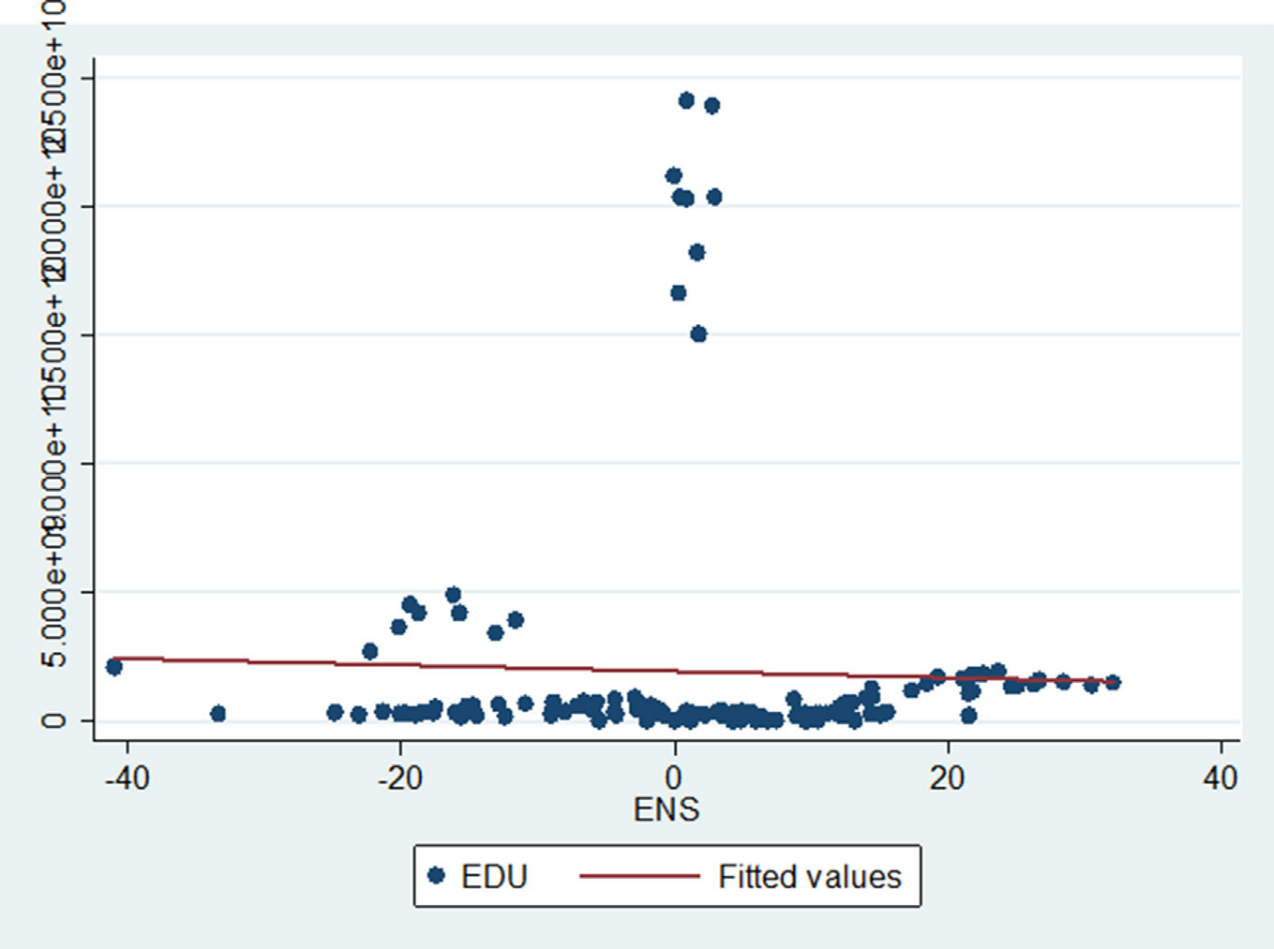
Showing the relationship between education and environmental sustainability.

The analysis indicated by Figures 1, 2, 3, 4, and 5 below reinforces the correlation results found in Table 6.

Figure 1 depicts the correlation between the Corruption Index (CORI) and environmental sustainability (ENS). It is moderately positive illustrating that as CORI increases (Transparency International (2020) asserts that an increase in the Corruption Index (CORI) implies the country becomes clean from corruption) we anticipate that ENS will also heighten.

Figure 2 indicates the association involving Corruption Ranking (CORRA) and environmental sustainability (ENS). It is moderately negative demonstrating that as CORRA increase we expect ENS to decline.

Figure 3 above shows the two-way relationship between income (GDP) and environmental sustainability (ENS). The link is lowly positive outlining that as GDP increases we expect ENS to also increase.

Figure 4 depicts the correlation between the ease of doing business (EDB) and environmental sustainability (ENS). It is moderately negative illustrating that as EDB increases (World Bank Group (2020) highlights that a high numerical EDB index implies the country's companies have a bad environment for doing business, and vice-versa) we anticipate that ENS will decline.

Figure 5 depicts the correlation between Education (EDU) and environmental sustainability (ENS). It is very lowly positive illustrating that as the current level of EDU increases we anticipate that ENS will decrease.

Figures 1, 2, 3, 4, and 5 only explain a one-to-one link. This paper will investigate an in-depth analysis of this association more when we apply the GMM approach that is dynamic and also add the effects of two or more variables on the relationships. Moreover, this article conducts Granger causality tests to prove causation. The outcomes of Granger causality tests are presented in the following section.

Table 7 presents the findings of the Granger Causality test outcomes generated through a bootstrap application technique. It is vital to note that the outputs show *W* (W-bar), Z (Z-bar), and *Ž* (Z-bar tilde) estimates. For the interests of this paper, I will only discuss that *Ž*(Z-bar tilde) estimates as according to Dumitrescu and Hurlin (2012) this estimate will be favoured when *N* is large and *T* is small. To explain (using Corruption Index), the null hypothesis that ENS does not Granger-cause CORI and ENS does not Granger-cause CORI are both rejected by the *Ž* (Z-bar tilde) statistic. This implies that corruption in Southern African states does cause the current level of environmental sustainability. Moreover, the current state of environmental sustainability in the Southern African countries does cause corruption. It is also imperative to note that when the Corruption Ranking variable is applied the null hypothesis that ENS does not Granger-cause CORRA and ENS does not Granger-cause CORRA are also both rejected. This implies that the causality results are congruent even when a different corruption indicator is employed. Thus to the best knowledge of this article causality involving the current state of environmental sustainability and corruption (and vice-versa) in the Southern African economies exists with feedback effects. This confirms earlier results produced by World Bank (2010) that corruption is greatly affecting sustainable growth of the African region. As well, Akhbari and Nejati (2019) also express that corruption in developing economies heightens environmental degradation by high emissions.

**Table 7:** Findings of pair-wise Granger Causality tests between variables and environmental sustainability.

Null hypothesis	*W*(W-bar) statistic [95% critical value]	Z (Z-bar) statistic [95% critical value]	*Ž*(Z-bar tilde) statistic [95% critical value]
CORI does not Granger-cause ENS	2.5020	4.2484 (0.3300)	0.7088 (0.3700)
ENS does not Granger-cause CORI	2.9238	5.4414 (0.2200)	1.0667 (0.2200)
CORRA does not Granger-cause ENS	4.5400	10.0127 (0.0900)	2.4381 (0.0900)
ENS does not Granger-cause CORRA	5.1039	11.6075 (0.0600)	2.9166 (0.0600)
GDP does not Granger-cause ENS.	3.1394	6.0510 (0.3100)	1.2496 (0.3100)
ENS does not Granger-cause GDP.	2.3793	3.9011 (0.3100)	0.6046 (0.4800)
EDB does not Granger-cause ENS	1.6107	1.7274 (0.6400)	-0.0475 (0.9500)
ENS does not Granger-cause EDB.	1.2370	0.6703 (0.8800)	-0.3646 (0.6200)
EDU does not Granger-cause ENS.	-	-	-
ENS does not Granger-cause EDU.	7.0969	17.2447 (0.0100)∗∗	4.6077 (0.0100)∗∗

Notes: ∗∗ indicates that the coefficients are significant at the 5% level of significance, respectively.

In addition, the null hypothesis that GDP does not Granger-cause ENS and ENS does not Granger-cause GDP is also rejected by the *Ž* (Z-bar tilde) statistic. This implies that GDP does Granger-cause ENS and ENS does Granger-cause GDP in the Southern African states thereby showing feedback effects of these relationships. More precisely, economic growth in southern African countries causes the current level of environmental sustainability, and vice-versa. Recent studies add more insights. For example, Abdouli and Hammami (2018) analysis of Middle Eastern and North African countries spanning 1990 to 2012 found bi-directional causality between economic growth and environmental degradation. Moreover, in ascertaining the causality among energy use, emissions and economic development for Pakistan, Mirza and Kanwal (2017) also highlights that economic growth and environmental quality develop bi-directional causality.

As well, the null hypothesis that EDB does not Granger-cause ENS and ENS does not Granger-cause EDB are also rejected by the *Ž* (Z-bar tilde) statistic. Thus, the findings in this context confirm that the ease of doing business Granger-cause environmental sustainability, and environmental sustainability also Granger-cause ease of doing business in Southern Africa. In critically elaborating on these findings Asongu and Odhiambo (2019) posits that ease of doing business particular factors such as start-up costs of business, energy provision challenges, lack of funding and high tax rates in Africa economies make it difficult for these countries to achieve a well-balanced, sustainable economic development platform. Ramachandran et al. (2009) also explains that most business models in African countries still lag behind in tackling the world's problems that have to do with among others the natural environment and poverty eradication.

The outcome with the null hypothesis that EDU does not Granger-cause ENS was not confirmed through the bootstrap process. The outputs also confirm that the null hypothesis which explains that ENS does not Granger-cause EDU is accepted by the *Ž* (Z-bar tilde) statistic thereby showing that the existing status of environmental sustainability does not Granger-cause education in Southern Africa. This finding is supported by Manteaw (2012) who confirms that education for sustainable development in Africa is little to non-existent as most academic settings, governments and also business contexts do not understand how sustainable development is promoted by the role of education. The following section presents findings on regression frameworks using static models.

Table 8 above presents the estimation results of the regression model 1 [Equation (1)]. The first concern relates to the heteroskedasticity of errors in the OLS framework. Thus, this paper implemented the Breusch-Pagan test (χ^2^) for heteroskedasticity. The results show that the estimate 232.63 with *p*-value of 0.000 is less than 5% hence the null hypothesis of homoscedasticity [that is error variances are all equal] is rejected and the alternative hypothesis of heteroskedasticity [that is error variances are not equal- (more particularly as the dependent variable –ENS increases, the variances increase or decrease)] is accepted. In this case, the POLS model is eventually rejected in favour of the Random Effect model.

**Table 8:** Findings of static panel data for regression 1: corruption index (CORI).

	Pooled Ordinary Least Square (POLS) Model		Random Effect Model		Fixed Effect Model	
	Coefficient	Standard Error	Coefficient	Standard Error	Coefficient	Standard Error
CORI	1.092267 (0.000)∗∗∗	0.1489519	0.4152181 (0.007)∗∗∗	0.154254	0.2649153 (0.104)	0.1618429
GDP	-0.0022555 (0.000)∗∗∗	0.0003814	-0.0017673 (0.019)∗∗	0.0007525	-0.0041631 (0.001)∗∗∗	0.0011752
EDB	-0.0011944 (0.974)	0.0370336	-0.0332544 (0.364)	0.0366359	0.0412009 (0.330)	0.0412009
EDU	4.96× 10^−11^ (0.806)	2.01× 10^−10^	4.02× 10^−10^ (0.335)	4.16× 10^−10^	1.02× 10^−9^ (0.082)∗	5.80× 10^−10^
R^2^	0.4636		0.2722		0.0239	
Wald (χ^2^)			10.02			
*F* statistic	30.03				3.59	
Breusch-Pagan test (χ^2^)			232.63 (0.000)∗∗∗			
Hausman test (χ^2^)					15.36 (0.0015)∗∗∗	
No. of observations	144	144	144	144	144	144

Notes: ∗∗∗; ∗∗ and ∗ indicate that the coefficients are significant at the 1%, 5% and 10% level of significance, respectively. Numbers in brackets are *p*-values.

In selecting between the Fixed Effects and Random Effects model estimates, a vital aspect is to find out if country effects are correlated with the independent and control (explanatory) variables in the regression. In contexts where there is no correlation, the Random Effects framework is reliable and hence more effective. Conversely, if there is evidence of correlation there is a possibility of excluded factor bias which compel adoption of the Fixed Effects estimates. Thus, it is apparent that when employing the Hausman Test the null hypothesis stipulates that the Random Effects model is the preferred framework while the alternative hypothesis confirms that the Fixed Effects is the appropriate model. The Hausman test outlined in Table 8 demonstrates that the estimate 15.36 has a *p*-value = 0.0015 which is less than 5% thereby favouring the Fixed Effect Model. As such, for the regression model (Equation [1]), I go ahead to estimate the dynamic panel data framework with fixed effects using the GMM approach.

Table 9 above presents the estimation outputs of regression model 2 [Equation (2)]. The findings illustrate that the Breusch-Pagan test (χ^2^) for heteroskedasticity support the Random Effect model than the POLS since the estimate 262.99 with a p-value of 0.000 is less than 5%. To choose between the Random Effect model and the Fixed Effect model the Hausman Test shows that the estimate 10.51 has a *p*-value = 0.0147 is less than 5% thereby necessitating acceptance of the Fixed Effect model. Thus, regression model 2 [Equation (2)] also compel this study to conduct a GMM analysis approach. The next section presents the regression outcomes generated by the GMM technique.

**Table 9:** Findings of static panel data for regression 2: corruption ranking (CORRA).

	Pooled Ordinary Least Square (POLS) Model		Random Effect Model		Fixed Effect Model	
	Coefficient	Standard Error	Coefficient	Standard Error	Coefficient	Standard Error
CORRA	-0.3099589 (0.000)∗∗∗	0.0416565	-0.1613043 (0.000)∗∗∗	0.0439541	-0.1031326 (0.031)∗∗	0.0473334
GDP	-0.0017139 (0.000)∗∗∗	0.0003452	-0.0016444 (0.015)∗∗	0.0006764	-0.0036776 (0.001)∗∗∗	0.0010699
EDB	0.0201881 (0.603)	0.0387638	-0.0069727 (0.851)	0.037052	0.0464953 (0.259)	0.0410138
EDU	6.5× 10^−11^ (0.656)	1.94 10^−10^	4.06× 10^−10^ (0.324)	4.11× 10^−10^	9.98×10^−10^ (0.084)∗	5.72× 10^−10^
R^2^	0.4680		0.3372		0.0091	
Wald (χ^2^)			16.31			
*F* statistic	30.56				4.16	
Breusch-Pagan test (χ^2^)			262.99 (0.000)∗∗∗			
Hausman test (χ^2^)					10.51 (0.0147)∗∗	
No. of observations	144	144	144	144	144	144

Notes: ∗∗∗; ∗∗ and ∗ indicate that the coefficients are significant at the 1%, 5% and 10% level of significance, respectively. Numbers in brackets are *p*-values.

Table 10 presents the two-step GMM short-run results to regression 1 (Equation [1]) that included that corruption index and regression 2 (Equation [2]) which included corruption ranking as the main independent variables, respectively. The outcomes show that lagged environmental sustainability indicates a positive relationship with environmental sustainability. In this case, a 1% increase in lagged environmental sustainability generates a 0.23% and 0.29% increase in environmental sustainability for regression 1 and 2, respectively. In this regard, by employing regression model (s) 1 and 2 with different corruption proxies, a unit rise in past environmental sustainability scenarios propels a rise of 0.23 and 0.29 percent respectively in environmental sustainability for Southern African countries. However, this finding is conflicts with Darkoh (2009) who express that Southern African countries' environments are being increasingly being affected by global warming, waste, pollution, desertification, deforestation, and loss of biodiversity.

**Table 10:** Two-step system-GMM findings with (a) corruption index (b) corruption ranking as the independent variables.

	Regression 1 (Corruption index)		Regression 2 (Corruption ranking)	
	Coefficient	Standard Error	Coefficient	Standard Error
$ENS_{it-1}$	0.2343317 (0.000)∗∗∗	0.0479454	0.2944566 (0.000)∗∗∗	0.0630367
CORI	0.106912 (0.000)∗∗∗	0.0151996	-	-
CORRA			-0.0562414 (0.000)∗∗∗	0.0106756
GDP	0.0011263 (0.064)∗	0.000607	-0.000359 (0.433)	0.0004579
EDB	-0.0564739 (0.000)∗∗∗	0.0026758	-0.0271819 (0.000)∗∗∗	0.0037096
EDU	7.3 × 10^−10^ (0.000)∗∗∗	2.05× 10^−11^	5× 10^−10^ (0.000)∗∗∗	2.74× 10^−11^
Constant	-2.428403 (0.514)	3.724972	11.97995 (0.000)∗∗∗	2.81339
Wald (χ^2^)	920.91 (0.000)		818.25 (0.000)	
Arellano-Bond test for AR (1) in first differences	z = -2.18 Pr > z = (0.029)∗∗		z = -2.03 Pr > z = (0.042)∗∗	
Arellano-Bond test for AR (2) in first differences	z = -0.88 Pr > z = (0.381)		z = -0.74 Pr > z = (0.461)	
Hansen test of overidentifying. Restrictions	Chi-square = 6.70 Prob > chi2 = (1.000)		Chi-square = 10.75 Prob > chi2 = (1.000)	

Notes: [1] ∗∗∗; ∗∗ and ∗ indicate that the coefficients are significant at the 1%, 5% and 10% level of significance, respectively. Numbers in brackets are *p*-values. [2] The null hypothesis of diagnostic statistical analysis shown in the table above are: (a) The Arellano-Bond test for autocorrelation: H_0_ = no autocorrelation; (b) The Hansen: H_0_ = the set of instruments is valid.

The findings also confirm that the corruption index (CORI) has a positive and significant relationship with environmental sustainability. In this case, as CORI increases (that is a decrease in corruption levels as countries become clean (Transparency International, 2020)) by 1%, then environmental sustainability also increases by 0.1069%. In addition, the results also highlight that corruption ranking (CORRA) has a negative and statistically significant link with environmental sustainability. In this regard, an increase in the rating of corruption of the country by 1% produces a decrease in environmental sustainability by 0.056%. Thus, for Southern African countries, it is quite evident that the harmonizing and congruent results contained in regression 1 and 2 by the GMM approach demonstrate that corruption practices degrade the natural environment in the short-term thereby in line with Wang et al. (2020) and Candau and Dienesch (2017) surveys. For this study, it is also important to note that CORI (0.1069%) has the greatest impact on ENS than CORRA (0.056%).

For regression one (with CORI) income show a positive and significant association with environmental sustainability for the Southern African countries although that relationship is negative and not significant in the case of regression 2 (with CORRA). Thus, since regression 2 results are not significant we can confirm that economic growth in Southern African countries is also not causing heightening environmental damage in the short-run as shown by regression 1 (with significant results). These findings agree with Ozcan, Tzeremes and Tzereme's (2020) exploration of 35 OECD economies from 2000 to 2014. The study spotlights that economic growth adds to these countries natural environmental performance. However, Sarkodie and Strezov (2018) survey of environmental Kuznets curve (EKC) and environmental sustainability in a few selected global countries validated the EKC and found that economic growth sectors of transportation, agriculture and service are the major drivers of environmental degradation in both developing and developed countries.

Moreover, the ease of doing business (EDB) demonstrates a negative and significant association with environmental sustainability for both regressions [1 and 2]. Hence, an increase of 1% of EDB (World Bank Group (2020) - high numerical EDB index implies the country's companies have a bad environment for doing business, and vice-versa) worsen environmental sustainability by 0.056% and 0.027% for Corruption Index (regression 1) and Corruption ranking (regression 2), respectively in the Southern African developing economies. Therefore, the easiness of doing business practices in Southern Africa seem to be causing environmental damage. More elaborately Haile (2007) and Manteaw (2012) ascertains that the difficulties in conducting business in Africa include tough regulatory requirements, stringent bureaucratic structures, little access to finance channels, weak tax systems, reduced protection to stakeholders in the private sector, less exposure to conduct international trade. On that note, such considerations ultimately affect a company's commitment to approaches that protect the natural environment in a negative way.

Furthermore, the level of education (EDU) illustrates a positive and significant connection with environmental sustainability in both cases [regression 1 and 2]. Thus, education is vital to support and improve environmental sustainability in the Southern African states. These outcomes agree with Balaguer and Cantavella (2018) who employed higher education data of Australia from 1950 to 2014 and contributes that education has been proven to improve the environment through emission reductions. Furthermore, Shumba et al. (2008) exploratory study on Zimbabwe's resettlement communities indicates that quality environmental education along with education for sustainable development implemented by participatory research was vital to ease tensions involving academic institutions and the community such that natural environmental projects were then permitted to start, and even improve.

The following section presents the GMM long-run findings of the study.

Table 11 presents the long-run GMM regression outputs from regression 1 and regression 2. Firstly, the results show that the corruption index and corruption ranking produce a negative and statistically significant association with environmental sustainability in the long-run. However, their diagnosis show conflicting results. To elaborate, on one hand, a 1% increase in corruption index (a decline in corruption levels as countries become clean) results in decreased environmental sustainability by a significant 0.127%. This finding contradicts with earlier research such as Biswas et al. (2012) survey on 100 economies; Sahli and Rejeb (2015) study on 21 MENA countries along with Akhbari and Nejati (2019) research on 61 countries who all found that corruption triggers environmental degradation. On the other hand, a 1% increase in corruption ranking (which is an increase in rating of country corruption) generates declines environmental sustainability by a significant 0.351%. Although the results are contradicting it is imperative to take into account that the corruption negative nature (becoming bad) as diagnosed by corruption ranking (-0.351%) on the natural environment is larger and seem to outweigh (nearly 3 times) the corruption positive nature (becoming clean) as diagnosed by corruption index (-12.7%).

**Table 11:** Findings of GMM long-run results with (a) corruption index (b) corruption ranking as the independent variables.

	Regression 1 (Corruption index)		Regression 2 (Corruption ranking)	
	Coefficient	Standard Error	Coefficient	Standard Error
CORI	-0.1274196 (0.004)∗∗∗	0.0444473		
CORRA		-	-0.350698 (0.000)∗∗∗	0.0723445
GDP	-0.2332054 (0.000)∗∗∗	0.0482653	-0.2948156 (0.000)∗∗∗	0.0632201
EDB	-0.2908056 (0.000)∗∗∗	0.0471652	-0.3216385 (0.000)∗∗∗	0.0601344
EDU	-0.2343317 (0.000)∗∗∗	0.0479454	-0.2944566 (0.000)∗∗∗	0.0630367

Notes: ∗∗∗; ∗∗; ∗ mean significant at 1%, 5% and 10% significance level, respectively. Numbers in brackets are *p*-values.

Moreover, both regressions [1 and 2] also demonstrate that both economic growth (GDP) show a significant and negative link with environmental sustainability. Thus a percentage rise in economic growth produces a 0.23% (with CORI regression) and 0.2948% (with CORRA regression) decline in environmental sustainability for the studied African countries. As such Saud et al. (2019) study on 59 Belt and Road Initiative (BRI) economies also argue that heightening economic growth results in decreased environmental quality. In addition, Chakravarty and Mandal (2016) research on the BRICS economies reveals that environmental degradation increases monotonically with heightening economic growth.

The results found from both regressions [1 and 2] also confirm that ease of business (EDB) show a significant and negative connection with environmental sustainability. Hence, when EDB heightens by 1% then 0.291% and 0.321% is the reduced proportion on the state of the natural environment. Thus, the long-run outcomes are also supporting earlier outcomes generated by the GMM short-run results in the previous section. In this situation, it is generally confirmed that doing business in Southern Africa is not assisting to lower environmental damage for sustainable development (Asongu and Odhiambo, 2019; Ramachandran et al., 2009).

Furthermore, in the long-term, the level of education for both regressions 1 and 2 illustrates that their relationship to environmental sustainability is evidently negative and statistically significant. Thus 1% increases in the level of education (EDU) leads to decreases in environmental sustainability estimated at 0.23% (with CORI regression) and 0.29% (with CORRA regression), respectively. A number of studies contradict with outcomes of this paper. For instance, Zimmerman and Weible (2017) argues that when students in rural areas of Mid-Atlantic USA (in Pennsylvania) where taught environmental education concepts they recognise the relevance of supporting environmental health and environmentally sound projects as that experience coupled with education passed gives them direction in solving community environmental problems. In the same vein, Zachariou et al. (2017) conducted a research in Greece (Viotia prefecture) and contributes that the teachers’ behaviour concerning environmental education is strongly associated with their conduct towards the natural environment and its challenges. As well, their environmental knowledge and information are strongly linked to positive conduct towards environmental education.

## 5 Implications of the study

This study produced essential findings on both causation and relationships between corruption and environmental sustainability. Both proxies of corruption; corruption index and corruption ranking Granger-cause the current state of environmental sustainability in Southern African countries, and that relationship is bi-directional implying that it has feedback impacts. The results also proved that in the short-run both indicators of corruption have a devastating and/or worsening effect on the existing state of environmental sustainability in the studied developing economies. In the long-run, the two proxies of corruption's effects on environmental sustainability are contradicting although the corruption negative (becoming bad) effect outweighs impacts linked to the corruption positive (becoming clean) influence. This demonstrates the detrimental influence of corruption on the natural environments.

As such, there is a need for Southern African governments to set-up relevant agencies that are independent and show zero-conflict on interests in green economy projects and other important roles that deal with natural environmental matters. As well, there is a need for governments to make sure that channels that support movement of environmental and green funding are clear, follow acceptable ethical guidance and access to financing from interest groups at both local and international levels are adequately prioritized. Moreover, establishing national agencies equipped with the responsibility to manage public funds in the adoption and implementation of environmental sustainability projects is equally important. Ideally, governments can also integrate monitoring systems in the central frameworks of environmental and green policies and projects. Such systems are imperative to promote anti-corruption security in the fundamental components of green and environmental policies and draw out improved coordination of major elements thereby doing away with any possible irregularities, obscurities, and inadequacies which may pave way for corruptible actions.

Business organizations have also a major role to play in efforts to mitigate corruption to maintain and/or improve environmental sustainability. For example, corporates can engage in open participation of green economy initiatives along with disclosure of vital natural environmental information (emissions, waste, energy, water) both at local and global contexts so that impartial and long-lasting green economy standards are accepted by relevant stakeholders. Best practices of corporate governance which put focus on anti-corruption activities in business operation are also critical in prospective green and/or environmental investment projects through adhering to high transparency and accountability values.

The findings of the paper also demonstrate that there is a bi-directional relationship between economic growth and the current state of environmental sustainability. As well, the short-run results indicate that when the corruption index was employed as the main independent variable [regression 1] income was found to heighten the current state of environmental sustainability in the explored Southern African states. Nonetheless, the long-run findings in the context of both corruption index [regression 1] and ranking [regression 2] illustrate that when economic growth increases the environmental sustainability of the Southern African economies declines. As such, it is vital to note that the Southern African economies are unable to maintain a good natural environmental scenario at high-income levels which is quite worrisome. Thus there is a need to introduce effective long-lasting green regulatory policies and strategies that ensure that green production of goods and services is attained and ultimately maintained. These countries should also integrate tough green legislations and promote the employment of environmentally-compatible technologies and clean development mechanisms to encourage local production. It is also vital for the government to do away with permitting extreme and/or dirty emission and waste-producing industries which damage the environment. Pollution industries can also be supported with inducements for adhering to acceptable environmental law and/or policy standards as well as taking into account natural environmental demands in both operational and decision-making levels.

As well, the outcomes of this paper put forward that ease of doing business develops a bi-directional association with environmental sustainability indicating that there are also feedback impacts concerning this link. In terms of the direction of association in both short-run and long-run ease of doing business (low ranking show improved easiness of implementing business activity) show a negative and significant connection with environmental sustainability which implies that organizations in Southern part of Africa have difficulties operating in the current state of environmental sustainability scenarios. Hence, there are no compatible policies involving business practice and natural environmental interests. In this case, there is a need to promote the diffusion of green technologies, improved green management structures and green best practices from companies that are from developed countries to the developing economies in Southern Africa since such firms can sustain harmonizing green standards and processes across many countries. As well, these states should also revise their international business policy since the extent of country liberalization along with trade openness is also fundamental in attracting superior cleaner technologies and policies.

Lastly, the outcomes of this research highlights that environmental sustainability in Southern African countries do not Granger-cause education. Moreover, the short-term and long-term direction of association is conflicting. In this context, the level of education (EDU) illustrates a positive and significant connection with environmental sustainability in the short-run but that link becomes significantly negative in the long-run. Therefore, it is apparent that human capital in these countries understands that their level of education is important to improve sustainability but surprisingly that relationship work against environmental sustainability improvement in the long-term. The reason to explain this situation could be the effects of corruption itself, poverty and political instability amongst other factors influencing the link between environmental sustainability and education. As such, creating contexts that improve education by improving its capacity, employment, knowledge, and emancipation in diverse sectors (through training and qualification programs) of the Southern African countries is vital to spearhead environmental sustainability. In this case, better green and environmental education is vital to transform individual mentality on how they relate to the natural environment which inevitably changes towards policies (social, economic and political) that are compatible with nature interests.

## 6 Conclusion

This paper investigated the influence of different indicators of corruption on the environmental sustainability of all the 16 countries that make up Southern Africa over the period 2010 to 2017. The article also deploys the Dumitrescu and Hurlin (2012) Granger causality tests and the Generalised Method of Moments (GMM) econometric techniques to establish causation and relationships respectively. Firstly, both indicators of corruption found a bi-directional link between corruption and environmental sustainability and that association was confirmed to worsen environmental sustainability in the short-rum although the relationship is contradicting in the long-run. Although the relationship is conflicting in the long-run the negative effect of corruption ranking surpasses the positive effect of corruption index by nearly 3 times thereby showing how detrimental corruptive actions are to the natural environment. Secondly, the research also establishes a bi-directional connection involving income and environmental sustainability. In the short-run corruption was ascertained to be increasing environmental sustainability but that relationship becomes negative in the long-term for these Southern African countries. Thirdly, ease of doing business develops a bi-directional link with environmental sustainability. Furthermore, in both short-run and long-run ease of doing business (low ranking show improved easiness of implementing business activity) show a negative and significant relationship with environmental sustainability for Southern African economies. Fourth, environmental sustainability is determined to not Granger-cause education. As well, education (EDU) demonstrates positive and significant association with environmental sustainability in the short-run but that connection is found to be significantly negative in the long-term. Overall, doing away with corruptible actions, greening economic growth strategies and policies, supporting green business policies and greening the education curriculum are fundamental processes vital to maintain proper environmental sustainability contexts in the Southern African region.

## Declarations

### Author contribution statement

F. Ganda: Conceived and designed the experiments; Performed the experiments; Analyzed and interpreted the data; Contributed reagents, materials, analysis tools or data; Wrote the paper.

### Funding statement

This research did not receive any specific grant from funding agencies in the public, commercial, or not-for-profit sectors.

### Competing interest statement

The authors declare no conflict of interest.

### Additional information

No additional information is available for this paper.
